# Proteomic Profile of Circulating Extracellular Vesicles in the Brain after Δ9-Tetrahydrocannabinol Inhalation

**DOI:** 10.3390/biom14091143

**Published:** 2024-09-10

**Authors:** Valeria Lallai, TuKiet T. Lam, Rolando Garcia-Milian, Yen-Chu Chen, James P. Fowler, Letizia Manca, Daniele Piomelli, Kenneth Williams, Angus C. Nairn, Christie D. Fowler

**Affiliations:** 1 Department of Neurobiology and Behavior, University of California Irvine, Irvine, CA 92697, USA; vlallai@uci.edu (V.L.); yenchc2@uci.edu (Y.-C.C.); jpfowler@uci.edu (J.P.F.);; 2 Yale/NIDA Neuroproteomics Center, Yale University, New Haven, CT 06511, USA; tukiet.lam@yale.edu (T.T.L.); rolando.milian@yale.edu (R.G.-M.); kenneth.williams@yale.edu (K.W.); angus.nairn@yale.edu (A.C.N.); 3 Department of Molecular Biophysics and Biochemistry, Yale University, New Haven, CT 06511, USA; 4 Keck MS & Proteomics Resource, Yale School of Medicine, New Haven, CT 06511, USA; 5 Bioinformatics Support Hub, Harvey Cushing/John Whitney Medical Library, Yale School of Medicine, New Haven, CT 06510, USA; 6 Department and Anatomy and Neurobiology, University of California, Irvine, CA 92697, USA; piomelli@hs.uci.edu; 7 Department of Psychiatry, Yale University, New Haven, CT 06511, USA

**Keywords:** cannabis, THC, extracellular vesicles, cerebrospinal fluid, proteomic

## Abstract

Given the increasing use of cannabis in the US, there is an urgent need to better understand the drug’s effects on central signaling mechanisms. Extracellular vesicles (EVs) have been identified as intercellular signaling mediators that contain a variety of cargo, including proteins. Here, we examined whether the main psychoactive component in cannabis, Δ9-tetrahydrocannabinol (THC), alters EV protein signaling dynamics in the brain. We first conducted *in vitro* studies, which found that THC activates signaling in choroid plexus epithelial cells, resulting in transcriptional upregulation of the cannabinoid 1 receptor and immediate early gene c-fos, in addition to the release of EVs containing RNA cargo. Next, male and female rats were examined for the effects of either acute or chronic exposure to aerosolized (‘vaped’) THC on circulating brain EVs. Cerebrospinal fluid was extracted from the brain, and EVs were isolated and processed with label-free quantitative proteomic analyses via high-resolution tandem mass spectrometry. Interestingly, circulating EV-localized proteins were differentially expressed based on acute or chronic THC exposure in a sex-specific manner. Taken together, these findings reveal that THC acts in the brain to modulate circulating EV signaling, thereby providing a novel understanding of how exogenous factors can regulate intercellular communication in the brain.

## 1. Introduction

Cannabis use has had a significant societal and health impact worldwide [1], spanning multiple cultures, economic levels, and medical fields, with both medicinal and recreational uses. Over the past few decades, global attitudes towards cannabis have changed, leading to legalization efforts in many countries, including throughout states in the US. Indeed, the increased availability of a variety of products, along with the marketing of cannabis as “natural” and thus “safe”, have been some of the main factors leading to increased consumption among the population [2]. This change has also sparked a thriving industry for the production and sale of cannabis products. However, challenges persist, including regulatory hurdles, concerns about dependence and abuse, and debates about its long-term health effects, either beneficial or negative. Thus, there is an urgent need to better understand the specific effects that cannabinoids may exert throughout the brain. The main psychoactive component in cannabis, Δ9-tetrahydrocannabinol (THC), has been shown to activate cannabinoid 1 (CB1) and cannabinoid 2 (CB2) receptors. CB1 is the main target for the psychoactive effects of cannabis [3,4]. Following aerosol exposure, maximal blood plasma THC concentrations are found within 30 min, and levels persist for at least 5–6 h post-intake [5,6,7]. Interestingly, differential patterns of CB1 receptor expression and plasma clearance rates have been found between males and females [3,5,8].

The choroid plexus is composed of epithelial cells localized within the brain’s ventricular system, which function to produce cerebrospinal fluid (CSF) and release various factors to modulate brain function [9,10,11,12,13,14], including extracellular vesicles (EVs). EVs have been identified as intercellular signaling mediators for the transport of RNA transcripts, proteins, including histones and enzymes, as well as other cargo [15,16,17,18,19,20,21,22,23,24,25], and EVs have been shown to be released from the choroid plexus [10]. Our previous research revealed the presence of active cholinergic signaling mechanisms within the choroid plexus, which provided a foundation for understanding how exogenous factors, such as nicotine, can directly influence the function of epithelial cells to mediate activation [26]. While the CB1 receptor has been shown to be expressed in the choroid plexus [27,28], along with enzymes involved in endocannabinoid formation [28], it has been unknown as to whether THC exposure leads to the modulation of epithelial cell function and, by doing so, causes EV release into the extracellular environment.

The overall goal of the present study was to investigate the effects of THC on EV signaling in the brain. Given the sex-specific differences in CB1 receptor expression and THC metabolism [3,5,8], both male and female rats were assessed. Further, with repeated dosing, metabolic clearance duration becomes extended, given the relatively long half-life of THC [8,29]. Thus, both acute and chronic dosing conditions were examined to assess whether differential effects were found based on the duration of exposure. These results reveal that THC induces the release of EVs with specific protein cargo and thereby provide a foundation for the identification of circulating biomarkers of sex-specific drug exposure.

## 2. Materials and Methods

### 2.1. Animals

Male and female Wistar rats were obtained from Charles River Laboratories (Kingston, NY, USA) and group-housed. Subjects were maintained in an environmentally controlled vivarium on a reverse 12 h:12 h light/dark cycle, and food and water were provided *ad libitum*. Experimental subjects were randomly assigned to treatment conditions for each sex. All procedures were conducted in strict accordance with ethical regulations outlined in the National Institutes of Health Guide for the Care and Use of Laboratory Animals and were approved by the Institutional Animal Care and Use Committee at the University of California, Irvine (Irvine, CA, USA).

### 2.2. Drugs

THC in ethanol solvent was generously provided by the NIDA Drug Supply Program (Bethesda, MD, USA). THC was prepared by evaporating ethanol under liquid nitrogen, and then the THC concentrate was dissolved in propylene glycol (50 mg/mL THC in PG) for vapor exposure or dissolved in cell culture medium at 50 μM for cell culture [30].

### 2.3. Experimental Design

#### 2.3.1. Primary Choroid Plexus Epithelial Cell Culture

Primary cultures of choroid plexus epithelial cells were prepared from dissected tissue of naïve, adult Wistar male and female rats (n = 6). Directly after decapitation, choroid plexus tissue was isolated from the third ventricle of the brain and then pooled across subjects to derive cell quantities for primary culture. Thereafter, cells were treated with collagenase Type II and TrypLE Express (Gibco/Invitrogen, Carlsbad, CA, USA). To obtain a monolayer, cells were plated in a modified chamber consisting of a silicon o-ring (Sealing Devices Incorporation, Landcaster, NY, USA), mounted on a cover glass (Warner Instruments, Holliston, MA, USA), and treated with poly-D-lysine coated to improve cell adhesion. Choroid plexus epithelial cell cultures achieved confluency by 2–3 d at 10,000 cells/cm^2^ with 10% FBS exosome-depleted serum and 1x pen-strep solution (Gibco/Invitrogen, Carlsbad, CA, USA). Culture media was changed every 2–3 days. Cell culture plates were maintained in a humidified 37 °C incubator containing 5% CO_2_. THC (50 μM) or cell culture medium vehicle was applied to the cells, and then cell tissue and cell culture medium were collected 90 min thereafter. EVs were extracted from cell culture using ExoQuick (Cat. EXOQ5A-1, System Biosciences, Palo Alto, CA, USA).

#### 2.3.2. Targeted RNA Expression Analysis

RNA was extracted from homogenized tissue derived from cell culture or EVs extracted from cell culture medium with TRIzol reagent (Ambion Life Technologies, Austin, TX, USA) via the manufacturer’s protocol. The quality of the RNA was determined by a NanoDrop 2000 spectrophotometer (ThermoScientific, Waltham, MA, USA). For each sample, 100 ng of total RNA was reverse transcribed into cDNA with the iScript cDNA synthesis kit (Bio-Rad Laboratories, Hercules, CA, USA). RT-qPCR was performed for c-fos (*Fos* gene, Cat #4331182), CB1 (*Cnr1* gene, Cat #4331182), mir-204 (Cat #4427975), and the housekeeping genes, b-actin (*Actb* gene; Cat #4331182) or U6 (RNU6; Cat #4427975) according to manufacturer’s parameters (Applied Biosystems, Waltham, MA, USA). Samples were tested in duplicate or triplicate (depending on the quantity of RNA available) and quantified with a CFX96 RT-qPCR system (Bio-Rad). Normalized gene expression (2^ΔCt^) was calculated with the equation 2^^(β-actin Ct − target mRNA Ct)^. For the microRNA assay, normalized mir-204 expression (2^ΔCt^) was calculated with the equation 2^^(U6 Ct − target miRNA Ct)^. All normalized values were multiplied by 1000. After samples were processed, group assignment was revealed to permit comparisons of the data. Data were analyzed by a two-sided *t*-test (GraphPad Prism, version 10, La Jolla, CA, USA). The criterion for significance was set at *p* < 0.05.

#### 2.3.3. Passive Vapor THC Administration

Rats were exposed to vaporized e-cigarette THC (50 mg/mL) during 1 h daily sessions for 1 day (acute) or 14 days (chronic) in LJARI vapor chambers (340 mm × 237 mm × 198 mm; LJARI, La Jolla, CA, USA) [31]. For each daily session, animals were exposed to THC in propylene glycol (PG) or PG vehicle at a rate of 1 puff (5 s programmed puff allowing for ~100 s of vapor presence in the chamber) every 5 min; this resulted in 12 total puffs per 1 h session.

#### 2.3.4. CSF Collection and EV Extraction from Rats

CSF was collected 30 min after the end of the session. Rats were placed under full anesthesia with isoflurane (3%) mixed with oxygen and placed in the nose cone of a stereotaxic apparatus. The back of the head was cleaned with chlorhexidine, followed by 70% ethanol. Using a surgical scalpel, the skin was cut down the midline, and connective tissue was removed to reveal the outer membrane covering the cisterna magna. A glass pipette was inserted into the outer membrane, and clear CSF (~100 μL) was collected in the pipette for each rat. Then, 100 µL of clear CSF from each subject was pooled together from 3 subjects according to the same sex and treatment group to create a total volume of 300 µL per sample (e.g., n = 3 subjects per sample). Immediately following extraction from the anesthetized rat, the pooled CSF was first filtered through a 0.2 µm cellulose acetate filter, followed by 15 s centrifugation and then immediately processed to extract EVs with SmartSEC™ Single columns (System Biosciences, Palo Alto, CA, USA) following the manufacturer’s instructions. The resulting samples were then stored at −80°C degrees until processing for mass spectrometry, as outlined below. This approach is ideal for the procedure as it isolates valuable samples with minimal volume requirements, eliminating the need for restorative buffers that can interact with mass spectrometry analyses.

#### 2.3.5. Label-Free Quantitative Proteomics Analyses via High-Resolution Tandem Mass Spectrometry

EVs were processed by the Keck MS & Proteomics Resource with tandem mass spectrometry analyses. The protein concentration of each sample was quantified, and 1.2 ug of protein was diluted and used for the mass spectrometer analysis. A label-free quantitative (LFQ) approach was implemented as described previously [32] with slight modifications to the sample preparation, as summarized below. Briefly, EVs were lysed with RIPA buffer containing protease and phosphatase inhibitor cocktail with ultra-sonication. EV debris was removed after centrifugation at 16,000× *g* for 10 min at 4 °C. Then, 175 μL of the supernatant was taken, and the protein was precipitated with Chloroform/MeOH/water (100:400:300 μL). Protein pellets were washed three times with cold methanol prior to drying by air. Dried protein pellets were resolubilized with an 8 M urea/0.4 M ammonium bicarbonate buffer, then reduced and alkylated with DTT and iodoacetamide, respectively. Proteins were then digested with trypsin (1:25 enzyme/protein ratio) overnight at 37 °C. Digested proteins were desalted with C18 reverse phase microspin columns (The Nest Group Inc., Southborough, MA, USA), and eluted peptides were dried using a SpeedVac. The total peptide amount was determined by nanodrop after reconstitution with 0.1% formic acid. Dilutions were made to ensure that an equal total amount of peptides (0.25 ug) were loaded on the column for the LC-MS/MS analyses. An equal amount of Pierce Retention Calibration Mixture (ThermoFisher, Waltham, MA, USA: Cat#: 88321) was spiked in all samples prior to injection for normalization when needed.

Label-Free Quantitation (LFQ) was performed on a Thermo Scientific Q-Exactive HFX mass spectrometer connected to a Waters M-Class ACQUITY UPLC system equipped with a Waters Symmetry^®^ C18 180 μm × 20 mm trap column and a 1.7 μm, 75 μm × 250 mm nanoACQUITY UPLC column (35 °C). Next, 5 µL of each digest (in triplicates) at 0.05 µg/µL concentration was injected in block randomized order. To ensure a high level of identification and quantitation integrity, a resolution of 120,000 was utilized for MS, and 15–20 MS/MS spectra were acquired per MS scan using HCD. All MS (profile) and MS/MS (centroid) peaks were detected in the Orbitrap. Trapping was carried out for 3 min at 5 µL/min in 99% Buffer A and 1% Buffer B prior to eluting with linear gradients that will reach 30% B at 140 min, 40% B at 155 min, and 85% B at 160 min. Two blanks followed each injection to ensure against sample carryover.

The LC-MS/MS data were processed with Progenesis QI software (Nonlinear Dynamics, version 4.2; Milford, MA, USA), with protein identification carried out using the in-house Mascot search engine (2.6). The Progenesis QI software performs chromatographic/spectral alignment (one run is chosen as a reference for alignment of all other data files), mass spectral peak picking and filtering (ion signal must satisfy the 3 times standard deviation of the noise), and quantitation of proteins and peptides. A normalization factor for each run was calculated to account for differences in sample load between injections as well as differences in ionization. The normalization factor was determined by calculating a quantitative abundance ratio between the reference run and the run being normalized, with the assumption being that most proteins/peptides are not changing in the experiment, so the quantitative value should equal 1. The experimental design was set up to group multiple injections (technical and biological replicates) from each run into each comparison set. The MS/MS spectra were exported as .mgf (Mascot generic files) for database searching. Mascot Distiller was used to generate peak lists, and the Mascot search algorithm was used for searching against the Swiss Protein database with taxonomy restricted to homo sapiens. Carbamidomethyl (Cys), oxidation (Met), phospho (Ser, Thr, Tyr), acetylation (Lys and Protein N-term), and deamidation (Asn and Asp) were entered as variable modifications. Two missed tryptic cleavages were allowed, precursor mass tolerance was set to 10 ppm, and fragment mass tolerance was set to 0.02 Da. The significance threshold was set based on a False Discovery Rate (FDR) of 1%. The Mascot search results were imported into the processed dataset in Progenesis QI software, where peptides were synched with the corresponding quantified features and their corresponding abundances. Protein abundances (requiring at least 1 unique peptide with a MOWSE score of >95% confidence) were then calculated from the sum of all non-conflicting peptide ion ID assignments for a specific protein on each run.

Protein quantitation Progenesis QI was carried out based on the sum of “non-conflicting” peptide matches. This process only uses peptides that are not also part of another protein hit to determine the protein abundance. Therefore, the protein abundance in a run is calculated from the sum of all the unique normalized peptide ion abundances corresponding to that protein. This provides a more robust, confident readout of protein abundance. Averages of protein abundances of biological replicates were conducted before the determination of fold changes between the conditions as calculated by taking the log base 2 of the ratios of conditions being compared. ANOVA (p) was calculated using a one-factor ANOVA calculation, which produces *p*-values to indicate the statistical significance of the difference in group abundance data. This between-subject calculation design assumes that the conditions are independent and, therefore, gives a statistical test of whether the means of the conditions are all equal. Values for the -log (ANOVA p) and log base 2 (ratio) were utilized in the Volcano Plot to gauge the significance of protein expression changes between the conditions. Data were screened for evidence of blood-specific proteins (RHD, RHCE, RHAG, KEL, ALAS2, CA5B, as determined by proteinatlas.org [33]), and we did not find detectable levels of these proteins, verifying the lack of blood contamination with the CSF collection process. The mass spectrometry proteomics data have been deposited to the ProteomeXchange Consortium via the PRIDE [34] partner repository with the dataset identifier PXD054211. Data were further compiled and visualized with QIAGEN Ingenuity Pathway Analysis software (version 24.0, Germantown, MD, USA).

## 3. Results

### 3.1. Effect of THC on Choroid Plexus Epithelial Cells in Primary Culture

**Figure 1:**
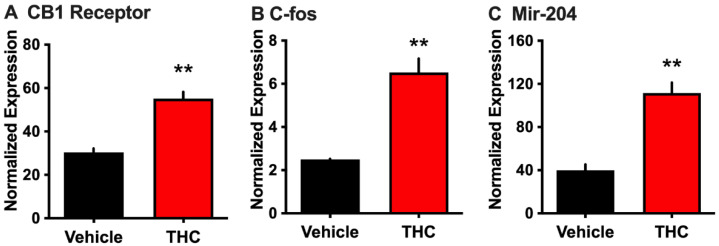
THC-induced changes in c-fos and CB1 receptor mRNA in primary choroid plexus cells and mir-204 in released EVs. Primary epithelial cell culture from the choroid plexus was examined following THC exposure (n = 6 rats). (**A**) THC induced a significant upregulation of cannabinoid receptor (CB1) mRNA. (**B**) Cellular activation was evidenced with THC exposure based on increased expression of c-fos mRNA. (**C**) From the cell culture medium, extracellular vesicles (EVs) were extracted, and THC treatment was found to induce a significant increase in the expression of EV-localized mir-204 as compared to control. ** *p* < 0.01. Data were normalized based on the expression of β-actin (*Cnr1* gene for CB1 and *Fos* gene for C-fos) or U6 (mir-204). Data represent mean normalized values ± SEM.

We first examined whether THC activates rat choroid plexus epithelial cells, which express CB_1_ receptors [27], and whether this could lead to the release of EVs containing miRNA-204, which is known to be localized in the choroid plexus [26]. Incubation of primary choroid plexus epithelial cell cultures with THC (50 μM for 90 min) resulted in a significant increase in *Cnr1* (CB_1_) transcription compared to vehicle (two-tailed *t*-test, t = 6.754, *p* = 0.0025) (Figure 1A). In parallel, THC exposure enhanced transcription of the activity-dependent immediate-early gene *c-fos* (two-tailed *t*-test, t = 6.236, *p* = 0.0034) (Figure 1B), indicating that THC can directly activate this cell population. To investigate if such THC administration would alter the release of RNA localized in EVs, the cell culture medium was then processed to extract EVs. Interestingly, THC treatment induced a significant increase in the amount of EV-associated mir-204 (two-tailed *t*-test, t = 6.390, *p* = 0.0031) (Figure 1C). Together, these data indicate that THC can modulate gene expression in the choroid plexus and enhance the extracellular release of miRNA localized in EVs.

### 3.2. Differentially Expressed EV Proteins in the Male CSF with Acute or Chronic THC Exposure

**Figure 2:**
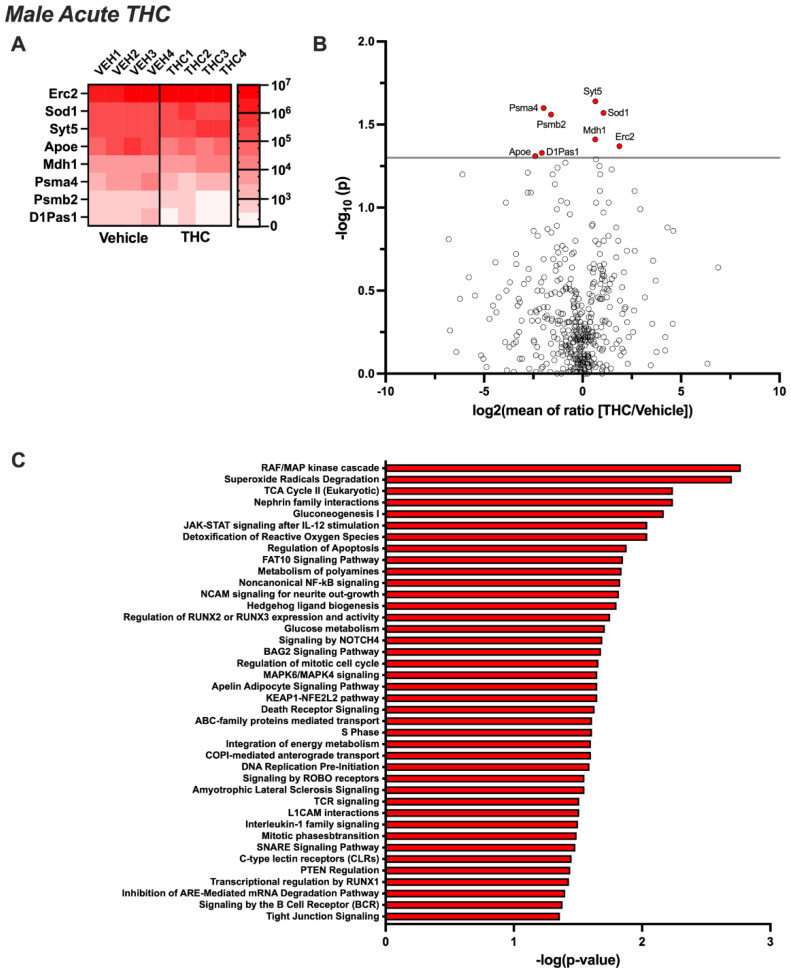
Acute THC-induced changes in the proteomic profile of CSF EVs from males. Male rats (n = 12/group) were permitted to inhale aerosolized THC or vehicle across a 1 h session. CSF was collected and pooled from 3 subjects for EV extraction (resulting in 4 samples per treatment group), and then proteomic analysis was performed. (**A**) The heatmap displays differentially expressed proteins for each sample analyzed per group (*p* < 0.05). (**B**) The volcano plot depicts the proteomic alterations within EVs isolated from the CSF based on significance set at *p* < 0.05. (**C**) Ingenuity Pathway Analysis reveals the networks governing the biological systems implicated in EV-localized proteins differentially regulated by acute THC vape exposure in male rats.

**Figure 3:**
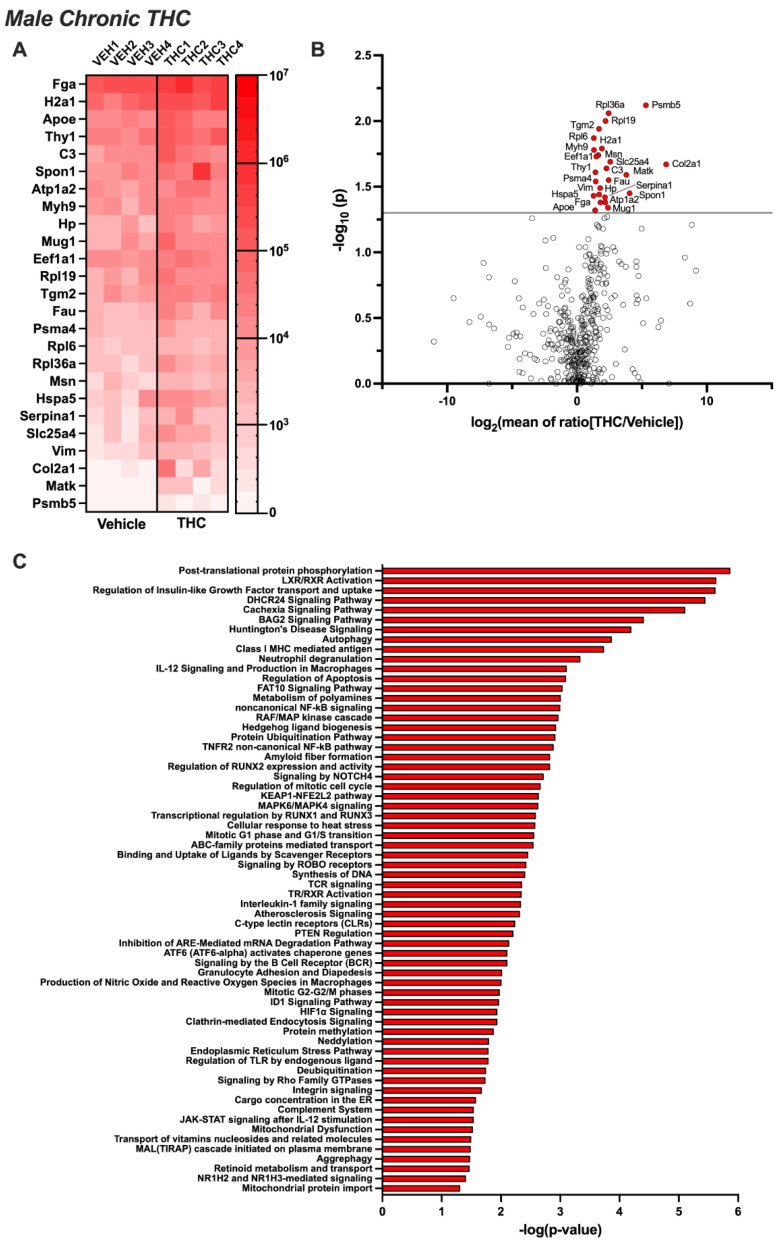
Chronic THC-induced changes in the proteomic profile of CSF EVs from males. Male rats (n = 12/group) were permitted to inhale aerosolized THC or vehicle across 1 h sessions for 14 consecutive days. CSF was collected and pooled from 3 subjects for EV extraction (resulting in 4 samples per treatment group), and proteomic analysis was performed. (**A**) The heatmap displays differentially expressed proteins for each sample analyzed per group (*p* < 0.05). (**B**) The volcano plot depicts the proteomic alterations within EVs isolated from the CSF based on significance set at *p* < 0.05. (**C**) Ingenuity Pathway Analysis reveals the networks governing the biological systems implicated in EV-localized proteins differentially regulated by chronic THC vape exposure in male rats.

Untargeted proteomic analysis of EVs from the CSF of male rats exposed acutely to aerosolized THC identified eight proteins that were differentially expressed compared to the control group (*p* < 0.05) (Figure 2A,B). Four proteins were significantly upregulated (Erc2, Mdh1, Sod1, and Syt5) and four were downregulated (Apoe, D1Pas1, Psmb2, and Psma4). Ingenuity Pathway Analysis revealed that these proteins were associated with cellular signaling pathways, immune function, biogenesis, metabolism, extracellular signaling interactions, apoptosis, cell cycle, protein transport, and disease states (Figure 2C). Next, we examined EV samples following 14 days of chronic exposure to aerosolized THC. Twenty-five proteins were found to be significantly upregulated, and none were identified as being downregulated (Figure 3A,B). The proteins that increased following chronic THC exposure in males included Apoe, Atp1a2, Col2a1, C3, Eef1a1, Fau, Fga, Hp, Hspa5, H2a1, Matk, Msn, Mug1, Myh9, Psma4, Psmb5, Rpl6, Rpl19, Rpl36a, Serpina1, Slc25a4, Spon1, Tgm2, Thy1, and Vim. Ingenuity Pathway Analysis showed that these proteins are associated with cellular signaling mechanisms, protein phosphorylation and expression, receptor activation, neurodegenerative disease states, immune signaling, apoptosis, metabolism, vesicle trafficking, and mitochondrial function (Figure 3C).

### 3.3. Differentially Expressed EV Proteins in the Female CSF with Acute or Chronic THC Exposure

**Figure 4:**
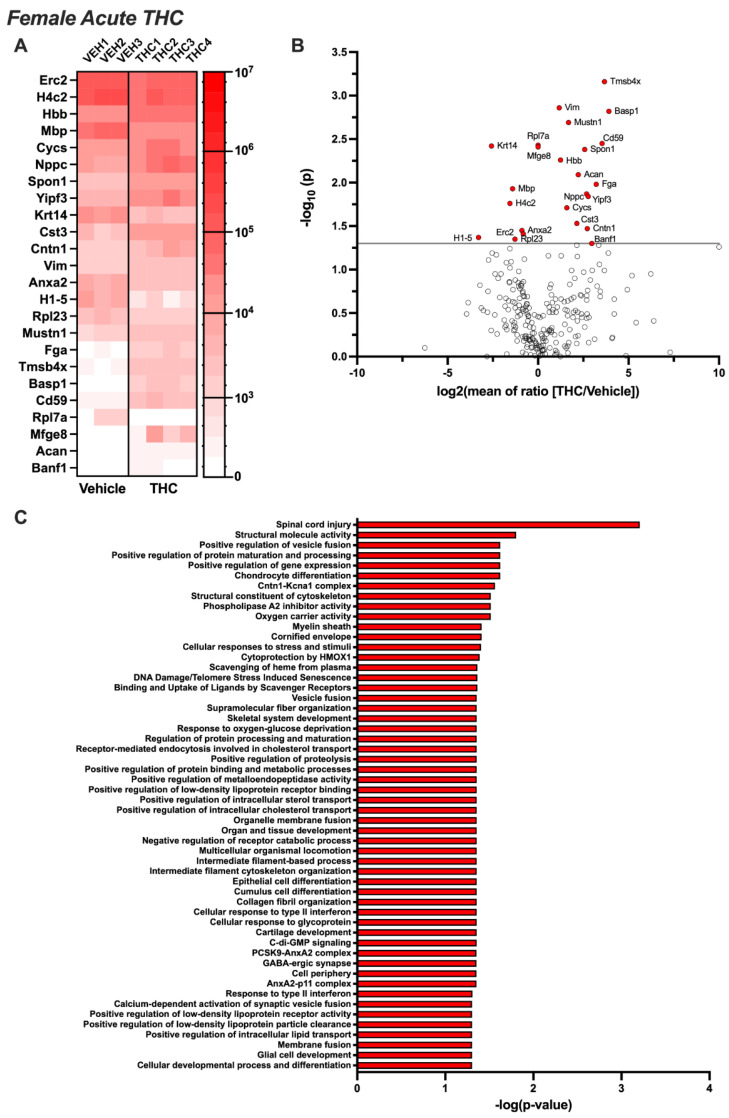
Acute THC-induced changes in the proteomic profile of brain EVs from females. Female rats (n = 9–12/group) were permitted to inhale aerosolized THC or vehicle across a 1 h session. CSF was collected and pooled from 3 subjects for EV extraction (resulting in 3–4 samples per treatment group), and then proteomic analysis was performed. (**A**) The heatmap displays differentially expressed proteins for each sample analyzed per group (*p* < 0.05). (**B**) The volcano plot depicts the proteomic alterations within EVs isolated from the CSF based on significance set at *p* < 0.05. (**C**) Ingenuity Pathway Analysis reveals the networks governing the biological systems implicated in EV-localized proteins differentially regulated by acute THC vape exposure in female rats.

**Figure 5:**
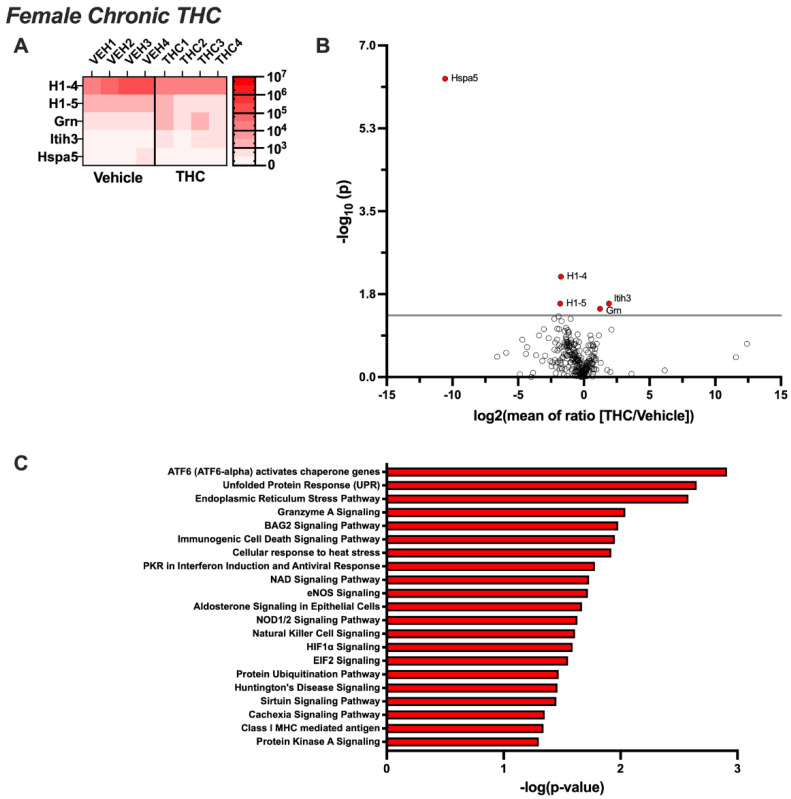
Chronic THC-induced changes in the proteomic profile of brain EVs from females. Female rats (n = 12/group) were permitted to inhale aerosolized THC or vehicle across 1 h sessions for 14 consecutive days. CSF was collected and pooled from 3 subjects for EV extraction (resulting in 4 samples per treatment group), and proteomic analysis was performed. (**A**) The heatmap displays differentially expressed proteins for each sample analyzed per group (*p* < 0.05). (**B**) The volcano plot depicts the proteomic alterations within EVs isolated from the CSF based on significance set at *p* < 0.05. (**C**) Ingenuity Pathway Analysis reveals the networks governing the biological systems implicated in EV-localized proteins differentially regulated by chronic THC vape exposure in female rats.

EVs extracted from the CSF of female rats exposed acutely to aerosolized THC led to the proteomic identification of 24 EV proteins that were differentially expressed compared to the control group (*p* < 0.05) (Figure 4A,B). Upregulated proteins included Acan, Banf1, Basp1, Cd59, Cntn1, Cst3, Cycs, Fga, Hbb, Mfge8, Mustn1, Nppc, Spon1, Tmsb4x, Vim, and Yipf3, whereas downregulated proteins included Anxa2, Erc2, H1-5, H4c2, Krt14, Mbp, Rpl7a, and Rpl23. Ingenuity Pathway Analysis revealed that these proteins are associated with neurodegenerative processes, protein phosphorylation, homeostatic pathways, inflammatory pathways, and synaptic signaling mechanisms (Figure 4C). Following 14 days of chronic THC or vehicle exposure, fewer proteins were differentially expressed, with only five identified (Figure 5A,B). While H1-5 remained downregulated, consistent with the acute exposure condition, H1-4 and Hspa5 also became downregulated. In contrast, Grn and Itih3 were significantly upregulated following chronic THC exposure in females. Ingenuity Pathway Analysis revealed that the proteins were associated with protein folding, stress-signaling pathways, neurodegenerative processes, inflammatory pathways, and intracellular signaling cascades (Figure 5C).

### 3.4. Pathway Analysis for Significant Targets

**Figure 6:**
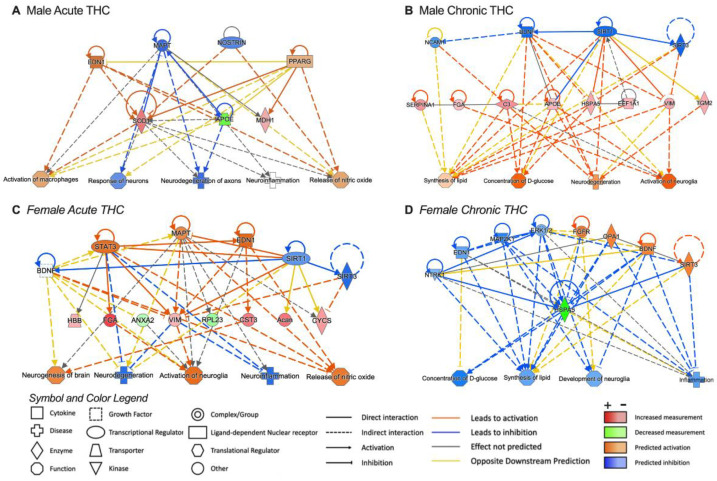
Pathway analysis for upstream regulators and downstream functions of proteins localized in circulating brain EVs with THC exposure. (**A**–**D**) Pathway analysis highlights upstream regulators of select identified proteins localized in EVs within the CSF following either acute THC exposure (males, (**A**); females, (**C**)) or chronic THC exposure (males, (**B**); females, (**D**)). The symbol and color legend are adapted from https://qiagen.my.salesforce-sites.com/KnowledgeBase/articles/Knowledge/Legend (accessed on 1 July 2024).

IPA software (version 24.0) was used to obtain insights into predicted upstream regulators and downstream outcomes on physiological function. For this analysis, we selected proteins demonstrating significant fold change after acute or chronic THC exposure (Figure 6). Interestingly, predicted upstream regulators included BDNF for most of the groups. Regarding the predicted downstream outcomes, activation of the nitric oxide pathway was associated with acute THC exposure in both male and female rats. Inhibition of neuroinflammation was also predicted following acute THC exposure, and this effect was more pronounced in female rats. Attenuated inflammatory processes were also predicted with chronic THC in females. Further, in males, a stronger effect was noted following chronic exposure to THC vapor inhalation for neurodegeneration and glial cell activation pathways.

## 4. Discussion

Proteins contained within EVs obtained from accessible biofluids can serve as relevant biomarkers of disease and have the further potential to reveal novel intercellular communication mechanisms. Here, we examined whether acute or chronic exposure to aerosolized THC would alter the proteomic cargo of EVs in male and female rats. We first found that THC can upregulate mRNA expression of the CB1 receptor and immediate early gene, c-fos, which demonstrates the functional effects of THC in modulating epithelial cell activity in the choroid plexus. Additionally, THC induced an increase in the release of EVs containing mir-204, a miRNA transcript abundant in the choroid plexus [26]. *In vivo* studies reveal that both acute and chronic exposure to THC modulates the protein cargo localized in EVs that are released into the extracellular environment of the brain. These effects of THC on EV protein cargo varied based on both the duration of exposure and the male or female sex. Together, these findings reveal an interaction between the effects of inhaled THC on the protein cargo of EVs circulating in the CSF based on sex, which thus provides insight into novel molecular mechanisms that may underly cannabinoid-induced alterations of the central nervous system.

### 4.1. Relevance of CSF-Derived Extracellular Vesicles

Emerging research has revealed the involvement of CSF-derived EVs in various pathologies. Investigations into neurological diseases, such as Alzheimer’s disease [35], Parkinson’s disease [36], and amyotrophic lateral sclerosis [37], have unveiled alterations in the cargo of CSF EVs, including changes in specific biomarkers associated with disease progression. Studies have also begun to reveal changes in EV cargo in patients using various drugs of abuse [38,39,40,41,42]. Of significance, a recent study from Ganesh and colleagues (2024) employed proteomics to uncover peripheral markers of neuropathology in young human cannabis users [43]. Among the 26 EV proteins identified as being significantly different in the blood of individuals with cannabis use disorder, several were associated with synaptic signaling, synaptic plasticity, and immune signaling [43]. In addition to similar pathway associations, three proteins were differentially regulated compared to the present findings. Specifically, in the current study, fibrinogen alpha chain (Fga) and complement C3 (C3) were upregulated with chronic THC vape exposure in males, whereas Fga and hemoglobin subunit beta (Hbb) were increased with acute THC exposure in female rats. Of interest Hbb has been found to be enriched selectively in the choroid plexus within the brain [33], whereas Fga and C3 expression has been associated with neurodegenerative conditions [44,45]. However, these THC-mediated changes in EV proteins contrast with the Ganesh study that found that these three proteins were downregulated in young cannabis users compared to control subjects [43]. These findings together highlight the challenges in studying the effects of drug use on EV signaling mechanisms given dynamics associated with (1) recency of drug exposure and subsequent impact on EV release and uptake mechanisms, (2) potential differential effects on brain versus blood EV signaling mechanisms, and (3) potential variations in the constituents of cannabinoid-containing products that may exert varying physiological effects. It also should be noted that while the present study analyzed EVs from CSF of male and female rats separately, the prior study [43] analyzed differential expression between EVs from plasma obtained from a group of 10 human subjects (4 females) with young onset cannabis use disorder (CUD) versus a control group that contained an equal number of age and sex-matched controls without CUD. In addition to being unable to determine sex-specific changes in expression, the prior study did not indicate whether the use of other drugs of abuse or comorbid health conditions were characteristic of the subjects analyzed, which also could have impacted EV cargo. These complex considerations related to examining EVs in humans highlight the need for discrete experimental manipulations in animal models to better disentangle the specific actions of drugs on extracellular signaling processes.

### 4.2. Interactions Based on THC Exposure Duration and/or Sex

In the present study, differential patterns of CSF EV protein cargo emerged with THC vapor inhalation based on sex. In males, both apolipoprotein E (Apoe) and proteasome 20S subunit alpha 4 (Psma4) were found to be downregulated following acute THC exposure but upregulated after chronic THC inhalation; in contrast, no differences in this protein were noted in females based on THC exposure. Indeed, ApoE has been previously found to be localized in EVs released from brain cells [46,47,48,49], thereby supporting this finding. Another protein, heat shock protein family A member 5 (Hspa5, also known as GPR78 or BiP), was found to exhibit an opposing sex-specific effect with chronic THC inhalation, in which expression was upregulated in male EVs but downregulated in female EVs. Interestingly, both Hspa5 and Apoe have been shown to be involved in viral production and immune response, in which ApoE contributes to viral assembly and Hspa5 interacts with the epitope-tagged envelope protein to promote internalization into cells as a chaperone protein [50,51,52,53,54]. It should be noted that EVs have long been described as being similar to envelope virus particles, given similarities in physical, chemical, and biological characteristics, which have been proposed to represent an innate biological mechanism to allow for intercellular communication across long distances in the brain and/or body [55]. An interactive effect was also found in protein expression based on the combined THC exposure duration and sex. Vimentin (Vim) was found to be upregulated in EVs with chronic THC exposure in males, as well as in acutely THC-exposed females. Vim is an intermediate filament involved in cell attachment and signaling but has also been implicated in the attachment of bacterial and viral pathogens to the host cell surface [56,57,58]. Thus, Vim could potentially function with Apoe and Hspa5 as described above to mitigate EV trafficking to specific cell populations and subsequently allow for EV integration into the target cell.

### 4.3. Putative Significance of Differentially Regulated Proteins

#### 4.3.1. Immune Function and Vesicular Trafficking

In addition to Vim, Hspa5, and Apoe, as discussed above, several other proteins associated with inflammation and innate immunity were found to be upregulated in a sex-dependent manner based on the duration of THC exposure. In females, chronic THC led to the increased EV expression of granulin precursor (Grn, also known as progranulin), which is an immune regulatory protein involved in lysosomal trafficking in which low levels are associated with various neurodegenerative diseases [59,60]. In males, acute THC exposure increased the EV expression of synaptotagmin V (syt5) and ELKS/RAB6-interacting/CAST family member 2 (Erc2, also known as Cast or Elks). Syt5 has been implicated in vesicular trafficking and exocytosis via Ca^2+^-dependent processes [61,62], whereas Erc2 appears to be involved in regulating neurotransmitter release at the membrane active zone [63,64], thereby suggesting that EVs may include membrane components from cells of origin. While it is currently unclear as to the function of the proteins when localized in EVs, these prior functional findings provide insight into avenues for future investigation with relevance to EV-specific mechanisms interacting with immune-associated functions.

#### 4.3.2. Epigenetic Regulators

Chronic THC inhalation in males induced an increase in transglutaminase 2 (Tgm2) expression in circulating brain EVs. Of significance, recent studies have shown that Tgm2 catalyzes protein post-translational modifications and mediates aminylation of several neurotransmitters, including serotonin, dopamine, and histamine, which leads to changes in chromatin organization to induce serotonylation, dopaminylation, and histaminylation of histone H3 [65,66,67,68]. In addition, acute THC inhalation in females resulted in the downregulation of EV-localized histone H1-5, and chronic THC inhalation in females led to downregulation in EV-localized histones H1-4 and H1-5. The H1 family of histones is involved in condensation of nucleosome chains and regulation of gene transcription with chromatin remodeling, nucleosome spacing, and DNA methylation. Further, H1-5 has been implicated in the progression of glioma and may also be involved in promoting cell survival [69,70], so such downregulation could present an anti-oncogenic function.

#### 4.3.3. Experimental Considerations and Limitations

In these studies, we did not conduct an analysis of the CSF to determine both the quantity and protein content of the EVs. While nanoparticle tracking analysis (NTA) could provide information regarding EV numbers, technical limitations preclude being able to examine the same EV CSF samples for both mass spectrometry and NTA together; specifically, the limited quantity of the original sample and concerns regarding protein degradation and sample dilution for an initial NTA analysis would negatively affect integrity for subsequent proteomic analysis. However, NTA and proteomic analyses could have been conducted in parallel from the collected CSF. In our pilot studies, we found that 50 μL of CSF was required to provide quantification with high rigor and reproducibility for NTA, which would have necessitated using half of each CSF sample. Therefore, future studies will need to be directed at determining whether the changes found were also reflective of THC-induced differences in EV density within the CSF. In further consideration of the dynamic nature of EVs in the organism, it should be noted that the changes in protein content may be reflective of either more/less (1) EVs being released, (2) packaging of proteins into each EV, and/or (3) EVs being taken up from the extracellular environment.

Some EVs have been shown to exhibit bidirectional movement across the blood–brain barrier, with an exchange between the brain CSF and systemic blood circulation [25,71,72,73,74]. Thus, while we provide evidence that THC can act directly on the choroid plexus to alter the release of EVs, we cannot conclude that all the findings evidenced by the proteomic data are due to choroid plexus-specific release. Thus, more targeted future studies will be necessary to elucidate the specific cell types contributing to all of the identified changes in EV proteins with THC treatment *in vivo*. *In vitro*, various other cell types have been shown to have the capacity to release EVs, including cancer cells, stem cells, immune cells, glial cells, and neuronal cells [25], and as such, future studies will need to examine the contribution of THC-mediated EV release from various potential cell types in the brain and periphery *in vivo*.

## 5. Conclusions

Taken together, the current findings provide evidence of sex-specific changes in brain-localized EV cargo in response to THC vape inhalation. Given our emerging understanding of the role of EVs in normal physiological and pathological conditions, these findings highlight the importance of understanding the impact of exogenous factors, such as THC, on extracellular signaling mechanisms.

## Data Availability

The mass spectrometry proteomics data have been deposited to the ProteomeXchange Consortium via the PRIDE [34] partner repository with the dataset identifier PXD054211.
